# Test–Retest Reliability of Functional Electromechanical Dynamometer on Five Sit-to-Stand Measures in Healthy Young Adults

**DOI:** 10.3390/ijerph18136829

**Published:** 2021-06-25

**Authors:** Daniel Jerez-Mayorga, Álvaro Huerta-Ojeda, Luis Javier Chirosa-Ríos, Francisco Guede-Rojas, Iris Paola Guzmán-Guzmán, Leonardo Intelangelo, Claudia Miranda-Fuentes, Pedro Delgado-Floody

**Affiliations:** 1 Faculty of Rehabilitation Sciences, Universidad Andres Bello, Santiago 7591538, Chile; daniel.jerez@unab.cl (D.J.-M.); cmiranda@unab.cl (C.M.-F.); 2 Grupo de Investigación en Salud, Actividad Física y Deporte ISAFYD, Universidad de Las Américas, Sede Viña del Mar 2531098, Chile; ahuerta@udla.cl; 3 Department Physical Education and Sports, Faculty of Sport Sciences, University of Granada, 18011 Granada, Spain; 4 Faculty of Rehabilitation Sciences, Universidad Andres Bello, Concepción 4030000, Chile; francisco.guede@unab.cl; 5 Faculty of Chemical-Biological Sciences, Universidad Autόnoma de Guerrero, Chilpancingo 39087, Mexico; ipguzman2@gmail.com; 6 Musculoskeletal Research Group, University Center for Assistance, Teaching and Research, University of Gran Rosario, Rosario 2000, Argentina; leonardo.intelangelo@gmail.com; 7 Department of Physical Education, Sports and Recreation, Universidad de La Frontera, Temuco 4811230, Chile; pedro.delgado@ufrontera.cl

**Keywords:** reproducibility, isokinetic, muscle

## Abstract

*Background:* The purpose of this study was to determine the reliability for the strength and movement velocity of the concentric phase from the five Sit-to-Stand (5STS), using three incremental loads measured by a functional electromechanical dynamometer (FEMD) in healthy young adults. *Methods:* The average and peak strength and velocity values of sixteen healthy adults (mean ± standard deviation (SD): age = 22.81 ± 2.13 years) were recorded at 5, 10 and 15 kg. To evaluate the reliability of FEMD, the intraclass correlation coefficient (ICC), standard error of measurement (SEM) and coefficient of variation (CV) were obtained. *Results*: Reliability was high for the 10 kg (CV range: 3.70–4.18%, ICC range: 0.95–0.98) and 15 kg conditions (CV range: 1.64–3.02%, ICC: 0.99) at average and peak strength, and reliability was high for the 5 kg (CV range: 1.71–2.84%, ICC range: 0.96–0.99), 10 kg (CV range: 0.74–1.84%, ICC range: 0.99–1.00) and 15 kg conditions (CV range: 0.79–3.11%, ICC range: 0.99–1.00) at average and peak velocity. *Conclusions:* The findings of this study demonstrate that FEMD is a reliable instrument to measure the average and peak strength and velocity values during the five STS in healthy young adults.

## 1. Introduction

The Sit-to-Stand (STS) is one of the most frequent motor gestures in daily life [1] and its effective realization depends on multiple determinants of both the environment and the physical capacity and strategies of the subjects [2]. Thus, the STS performance is considered a key factor in maintaining functional independence [3,4]. STS considers the vertical displacement of the center of mass from a low and stable position [5,6] to an elevated and less stable one, as well as being able also to consider different phases and events during its execution [7]. The STS transition is regarded as one of the most demanding daily physical activities [8], associated with a high level of energy expenditure [9] and joint stress [10]. The STS performance can be adversely affected by multiple clinical conditions [11,12], such as aging [13,14,15]. It has been reported that people who have difficulty in reaching the bipedal position are more likely to fall [16]. In this regard, although STS has been widely used in older adults with disabling conditions, it is important to consider that it has also been considered to assess the functional capacity of young adults [17,18] and children [19].

The muscular performance of the lower limb is fundamental for the functional capacity of the human being in different contexts [20,21], and STS has been proposed as a strategy for its assessment [22]. On the other hand, electromyography studies have described the synergic role of the lower limb muscle chain, highlighting the contribution of the quadriceps and hamstrings as executors of this movement [23]. In addition, the erector spinae and gluteal muscles are important when changing from the sitting to standing position in elderly persons [1]. Thus, STS-based exercise is considered one of the most effective resistance training modalities for improving lower extremity strength [24], especially for quadriceps, which, from the age of 50 years onwards, decreases by approximately 2–4% per year [25].

The STS evaluated through field tests corresponds to a valid and reliable alternative, which due to its simplicity, short administration time and minimal instrumentalization, is widely used in clinical studies [22,26,27]. However, it is important to keep in mind that the traditional expression of its raw result (time in seconds or number of repetitions) does not allow for other relevant indicators of muscular performance such as strength and velocity of execution, among other variables, to be obtained Therefore, instrumental methods that offer muscular performance assessment can contribute to a much more exhaustive functional evaluation.

Currently, the development of the functional electromechanical dynamometer (FEMD) (as opposed to the angular isokinetic devices) [28] allows systematizing and controlling multiple components of the load during the performance of natural movements, such as the range and velocity of movement, magnitude of resistance, control of strength exercised and type of muscle contraction (isometric, concentric and eccentric). In this regard, studies have assessed the reliability and validity [29,30,31,32] and used the FEMD [33,34,35,36,37,38] under different experimental modalities and muscle groups. However, studies that propose to assess the reliability of the STS test using this type of technology have not been previously developed.

According to the proposed background, the purpose of this study was to determine the reliability for the strength and movement velocity of the concentric phase from the five STS, using three incremental loads measured by a FEMD in healthy young adults.

## 2. Materials and Methods

### 2.1. Subjects

Sixteen subjects (eight women and eight men) volunteered to participate in this study (mean ± standard deviation (SD): age = 22.81 ± 2.13 years, body mass = 64.75 ± 10.60 kg, body height = 166 ± 16 cm, body mass index = 23.15 ± 2.48 kg/m^2^, body fat percentage = 25.81 ± 9.0%, skeletal muscle mass = 26.71 ± 6.01 kg). The inclusion criteria were (i) adult subjects (>18 years), (ii) both sexes, without distinction of race, and (iii) subjects free of physical limitations, health problems or musculoskeletal injuries that could compromise the evaluated performance. All subjects were informed of the procedures to be used and signed an informed consent form before initiating their participation in the study. The study protocol adhered to the tenets of the Declaration of Helsinki and was approved by the University of Granada Institutional Review Board (IRB approval: 619/CEIH/2018).

### 2.2. Study Design

A repeated measures design was used to evaluate STS strength and velocity using three incremental loads (5, 10 and 15 kg, respectively). Subjects attended two familiarization sessions, 48–72 h apart, and one week before the first experimental session took place. After two familiarization sessions, participants attended the laboratory on two separate days (at least 48 h apart) for two weeks. The same evaluator conducted all evaluations as they had experience of using the FEMD device. All sessions were performed at the same time of the day for each participant (±1 h) and under similar environmental conditions (~22 °C and ~60% humidity). The order of the loads were randomly established. This order was carried out in the two testing sessions.

### 2.3. Testing Procedures

All measurements were conducted at the faculty research laboratory, using a FEMD (Dynasystem, Model Research, Granada, Spain). The FEMD corresponds to a new technology that allows the evaluation and training of strength in humans, generating linear isokinetic velocities, dynamic modes (tonic, kinetic, elastic, inertial, conical) and static (isometric, vibratory), and allows for the evaluation and training through resistance/constant velocity and/or variables [39,40,41]. For each experimental session, the participants were invited to the laboratory after a four-hour fast, having rested and without having consumed caffeine 24 h before the experimental session. In addition, the participants wore sports clothing and footwear.

**Figure 1:**
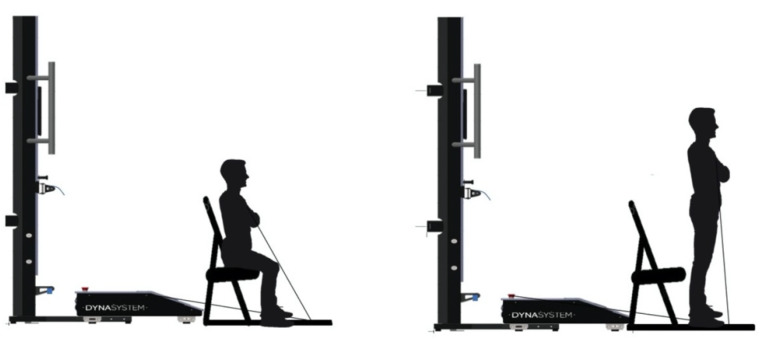
Set-up for STS using FEMD to measure movement velocity and strength.

All participants performed a standard warm-up on a low-load ergometer at a speed of 60 rpm for 10 min, 5 min of lower limb joint mobility and three to five submaximal (exercises that did not exceed a subjective intensity according to the modified Borg scale, <2–3 = light to moderate) repetitions of the STS protocol with the FEMD with a load of 5 kg. After this, the participants rested 2–3 min and were then evaluated through the FEMD by executing the five STS through three incremental loads of 5, 10 and 15 kg, respectively, performing five repetitions for each load with a 3 min rest interval between loads. In the five STS protocols, the subjects were seated in a rigid chair (height = 40 cm), with their arms across the chest and hip, knee and ankle joints at about 90°. From this position and at the command of the 3 2 1 countdown, participants stand and sit as fast as possible for five repetitions [42]. The linear displacement was measured by the FEMD rope attached to a harness on a vest used by each participant at the time of standing (Figure 1). The average strength (kg), peak strength (kg), average velocity (cm/s) and peak velocity (cm/s) of each stand was recorded during the concentric phase using the FEMD software.

### 2.4. Statistical Analyses

Descriptive data are presented as mean ± standard deviation (SD). The normal distribution of the data was confirmed using the Shapiro–Wilk test (*p* > 0.05). Paired sample *t*-test and standardized mean differences (Cohen’s d figure effect size (ES)) were used to compare the magnitude of the load between both testing sessions. The criteria to interpret the magnitude of the ES were as follows: null (<0.20), small (0.2–0.59), moderate (0.60–1.19), large (1.20–2.00) and very large (>2.00) [43]. Absolute reliability was assessed using the standard error of measurement (SEM) and coefficient of variation (CV), while relative reliability was assessed using the ICC, model 3.1 [44]. The following criteria were used to determine acceptable (CV ≤ 10%, ICC ≥ 0.80) and high (CV ≤ 5%, ICC ≥ 0.90) reliability [45]. Systematic bias was examined through Bland–Altman plots [46]. Finally, the Pearson’s product-moment correlation coefficient (Pearson’s r) was used to quantify the correlation of strength and velocity between both testing sessions. The criteria to interpret the magnitude of the r were null (0.00–0.09), small (0.10–0.29), moderate (0.30–0.49), large (0.50–0.69), very large (0.70–0.89), nearly perfect (0.90–0.99) and perfect (1.00) [43]. For all statistical calculations, a 95% confidence interval was used in the analysis. Statistical significance was accepted at *p* < 0.05. All reliability assessments were performed by means of a customized spreadsheet [47], while other statistical analyses were performed using the JASP software (version 0.14.1 http://www.jasp-stats.org accessed on 1 March 2021).

## 3. Results

No significant differences were found for average and peak strength during the different experimental conditions between both testing sessions, except for the average strength condition of 5 kg (*p* = 0.008; ES = 0.42) with a “small” ES magnitude. Absolute reliability provided stable repeatability for the average strength and peak strength condition, with a CV of less than 10% in almost all cases, except for the 5 kg condition in the average strength where the CV was 10.67%. Reliability was high for 10 kg (CV range: 3.70–4.18%, ICC range: 0.95–0.98) and 15 kg conditions (CV range: 1.64–3.02%, ICC: 0.99) at average and peak strength (Table 1).

No significant differences were found in the assessment of average and peak velocity between the test and retest using the STS (*p* > 0.05) for all conditions. Absolute reliability provided stable repeatability for the average and peak velocity condition, with a CV of less than 5% in all cases. Reliability was high for 5 kg (CV range: 1.71–2.84%, ICC range: 0.96–0.99), 10 kg (CV range: 0.74–1.84%, ICC range: 0.99–1.00) and 15 kg conditions (CV range: 0.79–3.11%, ICC range: 0.99–1.00) at average and peak velocity (Table 2).

**Figure 2:**
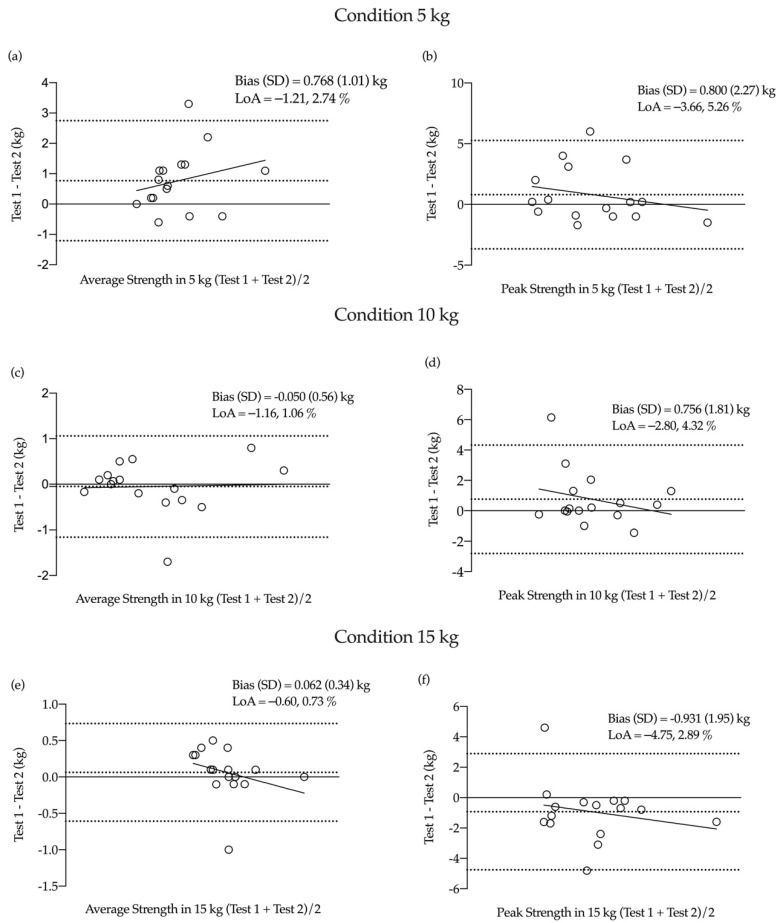
Bland–Altman plots of test–retest for average and peak strength. (**a**) average strength at 5 kg, (**b**) peak strength at 5 kg, (**c**) average strength at 10 kg, (**d**) peak strength at 10 kg, (**e**) average strength at 15 kg, (**f**) peak strength at 15 kg.

**Figure 3:**
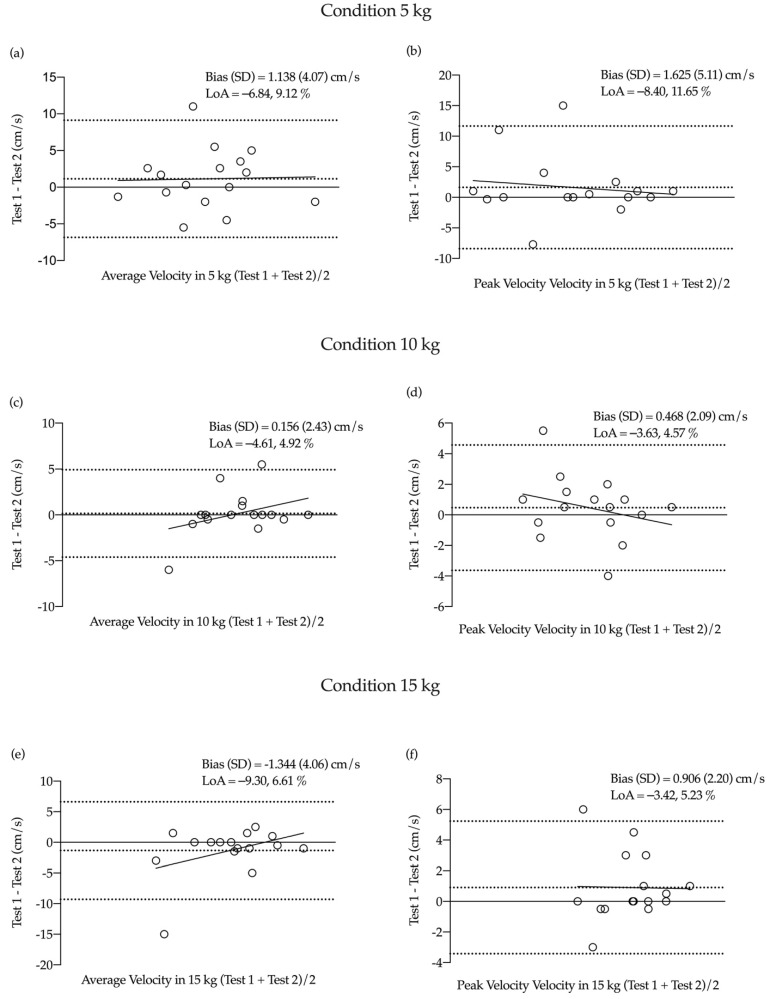
Bland–Altman plots of test–retest for average and peak velocity. (**a**) average velocity at 5 kg, (**b**) peak velocity at 5 kg, (**c**) average velocity at 10 kg, (**d**) peak velocity at 10 kg, (**e**) average velocity at 15 kg, (**f**) peak velocity at 15 kg.

Bland–Altman plots reveal a low systematic bias for average and peak strength (<0.80 kg) and velocity (<1.65 cm/s) during the 5, 10 and 15 kg conditions (Figure 2 and Figure 3).

**Figure 4:**
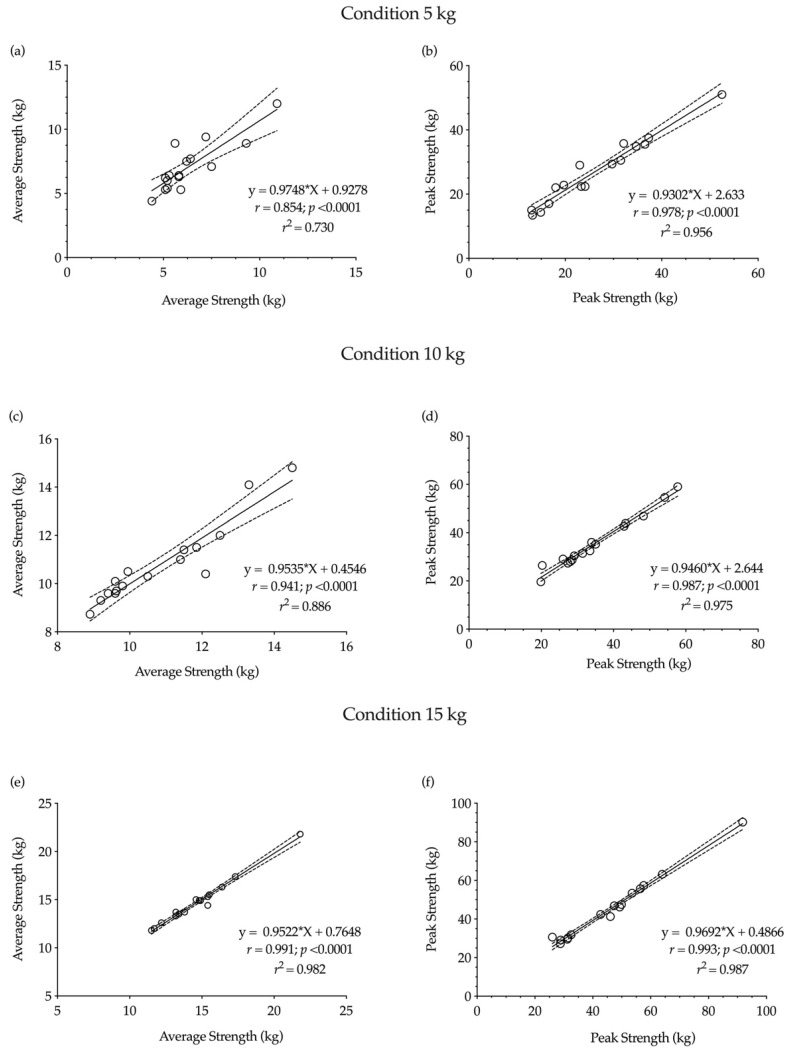
Relationship between average and peak strength during 5 kg, 10 kg and 15 kg conditions between both testing sessions. (**a**) average strength at 5 kg, (**b**) peak strength at 5 kg, (**c**) average strength at 10 kg, (**d**) peak strength at 10 kg, (**e**) average strength at 15 kg, (**f**) peak strength at 15 kg.

**Figure 5:**
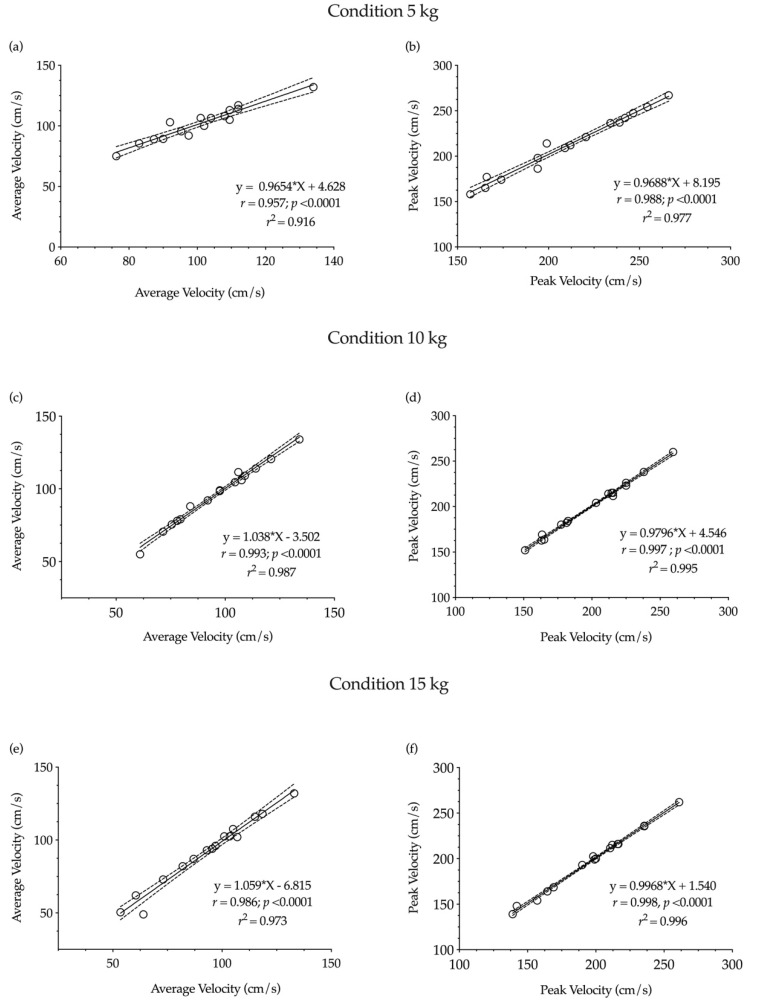
Relationship between average and peak velocity during 5 kg, 10 kg and 15 kg conditions between both testing sessions. (**a**) average velocity at 5 kg, (**b**) peak velocity at 5 kg, (**c**) average velocity at 10 kg, (**d**) peak velocity at 10 kg, (**e**) average velocity at 15 kg, (**f**) peak velocity at 15 kg.

Finally, the r magnitude for average and peak strength was from very large to nearly perfect during the different experimental conditions (*r* range = 0.854–0.993) and nearly perfect for average and peak velocity (*r* range = 0.916–0.996) (Figure 4 and Figure 5).

## 4. Discussion

The objective of this study was to determine the reliability for the strength and movement velocity of the concentric phase from the five STS, using three incremental loads controlled by a FEMD in healthy young adults. The main findings of this research show that there is “high” reliability for all conditions evaluated through the FEMD. These results show a stable repeatability for the protocols used (CV < 10%) [44].

Although this is the first study to evaluate the reliability of the STS using a FEMD, the reliability of other FEMD on the market has also been evaluated in recent years [29,30,31,32,48]. An example of this was the study by Campos et al. [48]. This study analyzed the validity and reliability at 0.25, 0.50, 0.75 and 1.0 m·s^−1^, using a FEMD (Haefni Health System 1.0^®^, Granada, Spain) and comparing it with a linear velocity transducer (LVT) (T-Force System^®^, Murcia, Spain). At the end of the study, an ICC of 0.99 was reported for both the concentric and eccentric phases, while the CV was higher for the velocity of execution (1.0 m·s^−1^ = CV 4.38%) [48]. In parallel, Chamorro et al. (2018) and Cerda et al. (2019) determined the reliability of a FEMD at the shoulder and hip joints, respectively [30,32]. The study by Chamorro et al. (2018) reported ICC values of 0.96 for 90° shoulder internal rotation and 0.94 for external shoulder rotation, and an ICC of 0.89 and 0.97 for shoulder internal and external rotation at 40°, respectively [32]. Likewise, to determine the validity and reliability of a FEMD in the hip joint, Cerda et al. (2019) used three isometric strength protocols on the hip abductor muscles. At the end of the study, the researchers reported a CV of 9.80, 6.60, and 5.64 for the side-lying, standing and supine positions, respectively [30]. Recently, Rodriguez-Perea et al. (2021) determined the validity and reliability of the FEMD (Dynasystem, Model Research, Granada, Spain) for assessing the velocity of movement, and the results indicated that the mean velocity values collected with FEMD and LVT were practically perfect correlations (r > 0.99) with low random errors (<0.06 m·s^−1^) [41]. In addition, the FEMD is reliable for assessing isometric and concentric strength of trunk flexors (CV range = 6.82–7.72) [29] and the effect of velocity on internal and external shoulder rotators (ICC: 0.81–0.98, CV: 5.12–8.27% SEM: 4.06–15.04 N) [39]. The previously referenced results are interesting since they allow us to compare the degree of reliability of the FEMD in different exercises, joint ranges, and muscle contraction types, showing that the FEMD is not only reliable for STS (CV < 10%), but also for other functional assessments.

The reliability of the tests used to assess physical fitness is influenced by the biological differences of the participants [49] and by of the variations that may occur in the instrument used to obtain the data [44]. In this sense, statistical analyses reported in some studies indicate that the reliability of the instruments is assured with an ICC value above 90% between two or more evaluations [44], while differences between participants can be reduced by including a familiarization period before data collection [29,36]. Therefore, the reliability achieved in the present study after multiple repetitions is due to two fundamental reasons: the accuracy of the FEMD used [30,32], and, on the other hand, the familiarization process that the participants had before data collection. Thus, it is important that when replicating this protocol, participants should start from a seated position, back straight, elbows bent and forearms close to the chest, while hips and knees should be bent at 90° (Figure 1). Any modification to this starting point could alter the reliability of the STS. In parallel and to maintain the reliability of the STS, it is important to consider the grip point, as it has been shown that distal and multi-joint grips have a lower ICC compared to proximal grips (ICC = 0.33) using a FEMD [50]. In the present study, the grip point was at the xiphoid process level; at this point, with the help of Velcro, it was anchored to the FEMD tension cable (Figure 1); this allowed fluid and natural execution of the movement, maintaining stable repeatability in all the repetitions.

The scientific literature has described the STS as a reliable test for assessing lower extremity muscle strength in young, healthy elderly and stroke patients [51]. A significant correlation has also been demonstrated with other functional mobility tests in patients with total knee replacement [52]. This test has also been used to observe the effect of oxygen therapy in patients with chronic obstructive pulmonary disease (COPD), showing that it is an excellent alternative to evaluate the functional capacity in this type of patient without the need for large implements [53]. In turn, the STS has been used as a standard functional balance test in children with cerebral palsy, proving to be a reliable test to assess this physical disability [54]. As it has become evident, the STS has functional applications in both healthy populations [51] and patients with various pathologies [52,53,54,55], proving to be a good indicator of strength, balance and functional capacity. Independently of this and increasing the reliability of the STS, influenced by the biological differences of the participants [49] or by the variations in the instruments used [44], We recommend including a familiarization session before data acquisition.

This study was not without limitations. The evaluation of strength and velocity during the concentric phase of the STS was only able to be evaluated in an initial condition of 5 kg because it was the minimum possible load to modulate in the FEMD. Therefore, it was not possible to determine the behavior of the average and peak velocity during an STS without load. On the other hand, the study was performed in a healthy population; future studies should study the behavior of strength and velocity in older adults where the STS has proven to be a valid tool to assess lower extremity muscle power [26] and the differences that may exist in the biomechanics of STS according to gender.

## 5. Conclusions

The findings of this study demonstrate that FEMD is a reliable instrument to measure the average and peak strength and velocity during the five STS in healthy young adults. Consequently, the trainer could have an additional alternative to record STS parameters and their progression in training programs, resulting in a useful tool for assessing the individual’s physical performance and physical capabilities.

## Figures and Tables

**Table 1:** Test–retest reliability of the average and peak strength in the 5STS using a FEMD.

	Condition	Session 1 (Mean ± SD)	Session 2 (Mean ± SD)	*p*-Value	ES (95% CI)	CV (95% CI)	SEM (95% CI)	ICC (95% CI)
Average Strength (kg)	5 kg	6.3 ± 1.7	7.1 ± 1.9	0.008	0.42 (−0.258–1.1459)	10.67 (7.88–16.52)	0.71 (0.53–1.11)	0.87 (0.66–0.95)
	10 kg	10.9 ± 1.6	10.8 ± 1.7	0.728	−0.03 (−1.041–0.92)	3.70 (2.73–5.72)	0.40 (0.30–0.62)	0.95 (0.86–0.98)
	15 kg	14.7 ± 2.5	14.8 ± 2.4	0.476	0.03 (−0.939–1.021)	1.64 (1.21–2.54)	0.24 (0.18–0.37)	0.99 (0.98–1.00)
Peak Strength (kg)	5 kg	26.3 ± 10.8	27.1 ± 10.3	0.180	0.08 (−0.905–1.056)	6.04 (4.46–9.35)	1.61 (1.19–2.49)	0.98 (0.94–0.99)
	10 kg	35.0 ± 11.3	35.7 ± 10.8	0.116	0.07 (−0.917–1.044)	4.18 (2.88–7.63)	0.40 (0.30–0.62)	0.98 (0.94–0.99)
	15 kg	46.1 ± 17.1	45.2 ± 16.6	0.075	−0.06 (−1.034–0.927)	3.02 (2.23–4.68)	0.24 (0.18–0.37)	0.99 (0.98–1.00)

SD, standard deviation; ES, Cohen’s d effect size ((higher mean–lower mean)/SD both); SEM, standard error of measurement; CV, coefficient of variation; ICC, intraclass correlation coefficient; 95% CI, 95% confidence interval.

**Table 2:** Test–retest reliability of the average and peak velocity in the 5STS using a FEMD.

	Condition	Session 1 (Mean ± SD)	Session 2 (Mean ± SD)	*p*-Value	ES (95% CI)	CV (95% CI)	SEM (95% CI)	ICC (95% CI)
Average Velocity (cm/s)	5 kg	100.8 (13.9)	102.0 (14.0)	0.281	0.08 (−0.894–1.066)	2.84 (2.10–4.40)	2.88 (2.13–4.46)	0.96 (0.90–0.99)
	10 kg	95.8 (19.8)	95.9 (20.7)	0.800	0.01 (−0.975–0.985)	1.80 (1.33–2.78)	1.72 (1.27–2.66)	0.99 (0.98–1.00)
	15 kg	93.0 (22.0)	91.7 (23.6)	0.205	−0.06 (−1.037–0.923)	3.11 (2.30–4.81)	2.87(2.12–4.44)	0.99 (0.96–1.00)
Peak Velocity (cm/s)	5 kg	210.8 (34.3)	212.4 (33.6)	0.223	0.05 (−0.933–1.027)	1.71 (1.26–2.65)	3.62 (2.67–5.60)	0.99 (0.97–1.00)
	10 kg	199.5 (31.2)	200.0 (30.6)	0.385	0.02 (−0.964–0.996)	0.74 (0.55–1.15)	1.48 (1.09–2.29)	1.00 (0.99–1.00)
	15 kg	196.6 (34.7)	197.5 (34.7)	0.121	0.03 (−0.954–1.006)	0.79 (0.59–1.23)	1.56 (1.15–2.42)	1.00 (1.00–1.00)

SD, standard deviation; ES, Cohen’s d effect size ((higher mean–lower mean)/ SD both); SEM, standard error of measurement; CV, coefficient of variation; ICC, intraclass correlation coefficient; 95% CI, 95% confidence interval.
